# Learning burnout and its association with perceived stress, social support, and the Big Five personality traits in Chinese medical students during the COVID-19 pandemic: a cross-sectional study

**DOI:** 10.1186/s12888-022-04453-6

**Published:** 2022-12-13

**Authors:** Simeng Wang, Honghe Li, Xin Chen, Nan Yan, Deliang Wen

**Affiliations:** 1 Institute for International Health Professions Education and Research, China Medical University, No. 77 Puhe Road, Shenyang North New Area, Shenyang, Liaoning 110122 People’s Republic of China; 2 Department of Epidemiology, School of Public Health, Dalian Medical University, No. 9 West Section of Lvshun South Road, Lvshunkou District, Dalian, Liaoning 116044 People’s Republic of China; 3 School of Medical Applied Technology, Shenyang Medical College, No.146 Huanghe Street, Yuhong District, Shenyang, Liaoning 110034 People’s Republic of China

**Keywords:** Learning burnout, Social support, Perceived stress, The Big Five personality traits, COVID-19, Medical students

## Abstract

**Background:**

Owing to the coronavirus disease 2019, medical learning burnout has attracted increasing attention in educational research. It has a serious negative impact on medical students and their service quality. This could impair the professional development of medical students; weaken their personal and professional quality; and lead to problems such as increased medical errors and reduced patient care quality and satisfaction. This study aimed to examine the effects of perceived stress, social support, and the Big Five personality traits on learning burnout among medical students.

**Methods:**

In November 2021, a cross-sectional survey was conducted at three medical universities in China. A self-administered questionnaire was distributed to 616 third- year students. Learning burnout, perceived stress, social support, and the Big Five personality traits (neuroticism, extroversion, openness, agreeableness, and conscientiousness) were anonymously measured. A total of 583 students were included in the final sample. Hierarchical linear regression was performed to explore the effects of perceived stress, social support, and Big Five personality traits on medical students’ learning burnout.

**Results:**

Perceived stress was positively associated with learning burnout (emotional exhaustion: *ß* = 0.577, *p* < 0.001; cynicism: *ß* = 0.543, *p* < 0.001; low professional efficacy: *ß* = 0.455, *p* < 0.001) whereas social support was negatively related with it (low professional efficacy: *ß* = -0.319, *p* < 0.001). Neuroticism had a positive effect on emotional burnout (*ß* = 0.152, *p* = 0.009). Extraversion (*ß* = -0.116, *p* = 0.006) and conscientiousness (*ß* = -0.363, *p* < 0.001) had a negative effect on low professional efficacy. Agreeableness negatively affected emotional exhaustion (*ß* = -0.181, *p* < 0.001) and cynicism (*ß* = -0.245, *p* < 0.001) and positively affected low professional efficacy (*ß* = 0.098, *p* = 0.008). The associated factors together accounted for an additional variance of learning burnout (emotional exhaustion: 39.0%; cynicism: 36.8%; low professional efficacy: 48.7%).

**Conclusions:**

Social support is a positive resource for fighting medical students’ burnout. Perceived stress was the strongest indicator of learning burnout. In addition to reducing perceived stress, developing extraversion, agreeableness, and conscientiousness should be included in burnout prevention and treatment strategies, particularly for medical students.

## Background

Burnout is a state of psychological distress and widely regarded as a significant work syndrome, originally used in the service industry [1]. Over time, the study of burnout has gradually extended to college students [2]. Learning burnout refers to students’ comprehensive performance such as emotional exhaustion, cynicism, and low professional efficacy caused by failure to meet academic requirements [3]. ‘Emotional exhaustion’ refers to the feeling of being emotionally overstretched and drained. ‘Cynicism’ refers to negative, indifferent, or overly detached reactions to others. ‘Low professional efficacy’ refers to a decrease in the sense of competence and success in academic learning [4].

Burnout in medical learning has attracted increasing attention in educational research [5]. Learning burnout is particularly common among medical students. Medical undergraduates must undertake at least to 5–7 years of medical training at a university before they can begin practice at a healthcare institution. Medical undergraduates typically receive more training than social science or business undergraduates. A previous systematic study showed that the observed incidence of academic burnout among medical students varied from 18 to 82%, depending on the tools used or socio-cultural context [6, 7]. According to recent information extracted from data published in English, the current burnout rate among medical students is 44.2%, with most studies using Maslach Burnout Inventory (MBI) tools [8]. In China, a systematic survey showed that 25.8–52.1% of medical students had a burnout level higher than average [9]. Learning burnout has a serious adverse impact on medical students and other special populations as well as on the quality of medical services. Meanwhile, it damages the professional development of medical students; weakens their personal and professional qualities; and leads to increased medical errors, lower quality of patient care, and lower patient satisfaction [10]. Therefore, this study aimed to explore the current level of learning burnout and its influencing factors among Chinese medical students.

The impact of a stressful event on an individual is partly determined by their perception of stress [11]. This is referred to as ‘perceived stress’, which is—the degree to which individuals consider situations in their lives to be stressful. Medical schools have unique stressors that go beyond the scope of college education [12]. In many medical schools, the environment itself presents pervasive stress [12]. A prospective study in the United States found that stress levels were associated with vulnerability to burnout among medical students [13]. Due to the complexity of learning tasks, medical students typically experience relatively higher levels of stress in terms of learning outcomes than students in other fields. Some studies have reported that students’ mental health deteriorates as the course progresses [14]. Furthermore, medical students are typically given more responsibility for human health and receive much greater attention from society than students in other disciplines, which can lead to increased stress. As medical students enter their third year of study, they may feel stressed by the addition of clinical specialty courses, which may lead to burnout. Owing to the coronavirus disease 2019 (COVID-19) pandemic that started in December 2019, most universities in China have adopted online teaching methods. In offline teaching, students consult teachers on time if they have questions, which is impossible in online teaching. Meanwhile, medical students are not allowed to participate in clinical internships, and the examinations of practicing physicians are postponed, which is a challenge.

Social support, defined as help and protection provided by others through formal or informal measures, is considered a protective factor in reducing burnout among medical students [15]. A Brazilian study of internal burnout found that seeking social support was not associated with burnout [16]. However, studies have pointed out that social support is a key factor in reducing the level of learning burnout [2]. Similarly, medical students with high levels of social support were less likely to experience burnout. During the COVID-19 outbreak, the Chinese government promptly implemented home quarantine and other public health emergency prevention and control measures nationwide to effectively control the spread of the epidemic [15]. In addition, the government and schools took steps to mitigate the impact of the pandemic on students, including providing daily necessities, increasing student subsidies, and psychological consultation [15]. Therefore, social support was particularly significant during this period, drawing widespread attention from the medical community.

In addition to the stress and social support mentioned above, studies have pointed out that personality traits play a role in burnout [17]. Moreover, it has been suggested that personality can help individuals avoid the known risk of burnout [18]. A 12-year longitudinal study of British medical graduates suggested that burnout was determined by personality [19]. A national study of Dutch residents noted that burnout risk was related to personality traits [20]. Personality is a unique mode of thinking, feeling, and behaving that persists over time and circumstances. Personality can be described by five major characteristics, popularly known as the Big Five personality traits [17]. The most common traits described in the Big Five personality framework are neuroticism, extraversion, openness, agreeableness and conscientiousness [21]. The psychological literature broadly suggests that individuals vary in their sensitivity to the risk of mood disorders depending on how much they rely on these five main personality traits [22]. A study of registered and practicing nurses in the United States found a negative relationship between extraversion and burnout [19]. A study of Romanian medical students found that students with high levels of neuroticism reported higher levels of emotional exhaustion [23]. However, at present, there has not been any research on the effects of the Big Five personality traits on learning burnout among Chinese medical students. Further research on this is required, especially considering the COVID-19 pandemic.

The basic starting point of social cognitive theory is that human activities are determined by the interaction of three factors: individual behavior, individual cognition and other individual characteristics, and the individual's external environment [24]. Following this theory, this study investigated learning burnout among third-year undergraduates majoring in clinical medicine at three medical schools under the impact of COVID-19. Furthermore, this study aimed to determine the effects of perceived stress, social support, and the Big Five personality traits on medical students’ burnout.

## Methods

### Research design and sample

A cross-sectional survey was conducted in November 2021, recruiting third-year medical undergraduates from three medical schools: China Medical University, Dalian Medical University, and Shenyang Medical College. Using a power of 80%, a confidence level of 95%, and a margin of error of 5%, the estimated sample size was set to 383 using the following formula: X = Z_a/2_^2^ *p*(1 - p)/MOE^2^ [25, 26]. To account for incomplete questionnaires (i.e. up to 20% incomplete), the minimum number of participants were determined to be 479. Based on the sample size obtained in the early stages and the respective situations of the three universities, several classes were randomly sampled from the third-year undergraduate students majoring in clinical medicine at each university in the same proportion; a total of 616 medical students were recruited, and 603 questionnaires were collected. Twenty questionnaires had obvious errors and incorrect or incomplete answers to the polygraph questions. Effective responses were received from 583 students, with an effective response rate of 94.64%. This study adopted the method of an online survey and anonymous filling and did not involve private and sensitive topics. The questionnaire survey was conducted through an online survey platform called ‘Questionnaire Star’, and the contact person sent a direct link to the students who volunteered to fill out the questionnaire. The questionnaire system provided lucky rewards to encourage students to participate in the research and ensure the quality of the questionnaires. This study complied with the relevant requirements of the ethics committee.

### Measuring instruments

#### Maslach Burnout Inventory-Student Survey (MBI-SS)

The MBI is currently the most commonly used burnout scale and includes three subscales: exhaustion, cynicism, and low professional efficacy [8]. Exhaustion has been examined in different countries using the MBI-SS, which is tailored to students and consists of 15 items assessing learning burnout in higher education. The assessment was answered by the students on a 7-point Likert scale ranging from 0 = *never* to 6 = *always*. The MBI-SS showed satisfactory psychometric characteristics across all three dimensions for student populations in different countries [27–30]. Cronbach’s a for emotional exhaustion, cynicism, and academic inefficacy were 0.91, 0.868, and 0.908, respectively.

#### Perceived Stress Scale (PSS-10)

To measure whether respondents found stressful situations in their lives, the Turkish adaptation of the PSS-10 was used [31, 32]. The PSS-10 includes 10 items on the frequency of stressful events that occurred in the month prior to the research, which were assessed on a 5-point Likert scale (0 = *never* to 4 = *very often*). The internal reliability of the Turkish version is adequate (0.70) [31]. Cronbach’s a for this sample was 0.82.

#### Perceived Social Support Scale (PSSS)

The PSSS is a self-report scale comprising 12 items measuring perceived support from three domains: family, friends, and significant others [33]. Participants who completed the PSSS were asked to indicate how much they agreed with the project on a 7-point Likert scale, ranging from 1 (*very strongly disagree*) to 7 (*very strongly agree*). Higher total scores indicated higher levels of perceived social support. The translated version of this scale is widely used in China. The total scale and three subscales have good internal consistency and reliability [34]. Cronbach’s a for this sample was 0.98.

#### NEO Five-Factor inventory (NEO-FFI)

The Chinese version of the NEO-FFI was used to measure the Big Five domains, namely neuroticism, extraversion, openness, agreeableness, and conscientiousness. Neuroticism is characterized by frequent worry, and greater emotional instability, including more frequent and intense negative affects [35]. Extroversion is characterized by being energetic, and sociable [36]. Openness is characterized by greater acceptance of novel things and easier access to different emotional experiences [37]. Agreeableness is characterized by friendly and empathic behavior [22]. Conscientiousness manifests itself in self-disciplined, orderly, and planned behaviors [38]. It consists of 60 items (12 per domain), with answers on a 5-point Likert-type scale ranging from 1 (*completely disagree*) to 5 (*completely agree*). The reliability estimates for each domain were acceptable (0.63–0.85) and consistent with other Chinese studies concerning the questionnaire used [39]. Cronbach’s alphas for neuroticism, extraversion, openness, agreeableness, and conscientiousness were 0.818, 0.759, 0.654, 0.582, and 0.836, respectively. The subscales have acceptable internal consistency.

### Demographic characteristics

Demographic information regarding gender, place of residence, only-child, parental education status and exercise were obtained. The place of residence was categorized as a city or village. Education was categorized as junior high or lower, senior high school, and junior college or higher.

### Statistical analysis

Statistical analysis was performed using the Statistical Package for the Social Sciences (SPSS, version 20.0). A two-tailed probability value of < 0.05 was considered statistically significant. The inspection of histograms and analysis of skewness and kurtosis values for the study variables revealed that the data were approximately normally distributed. The descriptive statistics of the study variables were represented by mean value, standard deviation (SD), number (N), and percentage (%), as appropriate. An independent samples t-test and one-way analysis of variance (ANOVA) were performed to examine the distribution of learning burnout among the demographic factors. Multiple comparisons were performed when one-way ANOVA was significant. The Pearson’s correlation coefficient was performed to test the correlation between continuous variables. Hierarchical linear regression analysis was performed to explore the relationship between perceived stress, social support, the Big Five personality traits, and learning burnout after adjusting for covariates, which were related to learning burnout (emotional exhaustion, cynicism, and low professional efficacy) in univariate analysis (*p* < 0.05). Data including R^2^, adjusted R^2^, R^2^ changes, F, standardization regression coefficients (*ß*), and *p*-values were provided for each step in the regression model. Tolerance (> 0.10) and variance inflation factors (< 5) were tested for multicollinearity.

## Results

### Characteristics of participants

Of the 616 questionnaires sent, 583 (94.64%) were considered valid and suitable for analysis. Table 1 shows the demographic characteristics. Females represented 55.4% of the sample, and 59.2% of the students were from the city. The educational status of most of the students’ fathers was junior college or higher, and that of their mother’s was junior high or lower. Most students did not exercise (91.9%). Male medical students were more likely to suffer from cynicism than female students (*p* < 0.05). Students who lived in villages were more likely to suffer from low professional efficacy than those who lived in cities (*p* < 0.05). Students whose parents had a junior high education or lower were more likely to suffer from low professional efficacy than those whose parents had a junior college education or higher (*p* < 0.05).

**Table 1:** Demographic variables and differences in learning burnout (*N* = 583)

	N (%)	Emotional exhaustion (Mean ± SD)	F/t^c^	*p*	Cynicism (Mean ± SD)	F/t^c^	*p*	Low professional efficacy (Mean ± SD)	F/t^c^	*p*
**Demographic variables**										
**Gender**			0.70	0.482		2.96	0.003*		-1.38	0.167
Male	260 (44.6)	13.01 ± 6.51			9.73 ± 5.08			13.93 ± 7.27		
Female	323 (55.4)	12.63 ± 6.56			8.48 ± 5.10			14.76 ± 7.20		
**Place of residence**			0.22	0.824		-1.72	0.086		-2.28	0.023*
City	345 (59.2)	12.85 ± 6.81			8.73 ± 5.21			13.84 ± 7.53		
Village	238 (40.8)	12.73 ± 6.13			9.47 ± 4.97			15.20 ± 6.72		
**Only-child**			1.51	0.132		0.80	0.423		-1.51	0.129
Yes	325 (55.7)	13.16 ± 6.82			9.19 ± 5.34			13.99 ± 7.48		
No	258 (44.3)	12.34 ± 6.15			8.85 ± 4.84			14.90 ± 6.91		
**Father's education status**			0.08	0.924		1.04	0.354		4.82	0.008*
Junior high or lower	226 (38.8)	12.75 ± 6.04			9.39 ± 4.99			15.54 ± 6.63^a^		
Senior high school	108 (18.5)	12.64 ± 6.18			9.03 ± 4.71			13.95 ± 6.41		
Junior college or higher	249 (42.7)	12.92 ± 7.12			8.71 ± 5.40			13.54 ± 7.96^b^		
**Mother's education status**			0.71	0.492		0.91	0.403		4.45	0.016*
Junior high or lower	246 (42.2)	12.80 ± 6.01			9.36 ± 4.97			15.26 ± 6.62^a^		
Senior high school	114 (19.6)	12.20 ± 5.85			8.68 ± 4.46			14.57 ± 6.65		
Junior college or higher	223 (38.3)	13.10 ± 7.38			8.86 ± 5.59			13.35 ± 8.03^b^		
**Exercise**			0.27	0.778		-0.13	0.898		1.96	0.055
No	536 (91.9)	12.82 ± 6.61			9.03 ± 5.12			14.53 ± 7.35		
Yes	47 (8.1)	12.55 ± 5.77			9.13 ± 5.16			12.81 ± 5.63		

^a,b^Calculated by Dunnett T3 (unequal variances), mean scores for learning burnout with unequal superscripts differ significantly at the *p* < 0.05 level

^c^Independent sample t-test and one-way ANOVA were used

### Correlations among continuous variables

Table 2 shows that learning burnout was statistically significantly positively correlated with perceived stress (*r* = 0.462 to 0.577; *p* < 0.001) and neuroticism (*r* = 0.410 to 0.540; *p* < 0.001), and negatively associated with social support (*r* = -0.601 to -0.139; *p* < 0.001), extroversion (*r* = -0.543 to -0.283; *p* < 0.001), openness (*r* = -0.423 to -0.313; *p* < 0.001), agreeableness (*r* = -0.489 to -0.170; *p* < 0.001), and conscientiousness (*r* = -0.629 to -0.353; *p* < 0.001).

**Table 2:** Descriptive statistics and zero-order correlations (Pearson’s r) among study variables

Variables	1	2	3	4	5	6	7	8	9	10
1. Emotional exhaustion	1	0.803***	0.094*	0.577***	-0.139***	0.540***	-0.283***	-0.313***	-0.436***	-0.353***
2. Cynicism		1	0.134***	0.543***	-0.253***	0.521***	-0.313***	-0.380***	-0.489***	-0.424***
3. Low professional efficacy			1	0.462***	-0.601***	0.410***	-0.543***	-0.423***	-0.170***	-0.629***
4. Perceived stress				1	-0.407***	0.792***	-0.539***	-0.452***	-0.471***	-0.615***
5. Social support					1	-0.366***	0.538***	0.453***	0.230***	0.582***
6. Neuroticism						1	-0.538***	-0.499***	-0.525***	-0.600***
7. Extroversion							1	0.441***	0.182***	0.626***
8. Openness								1	0.421***	0.662***
9. Agreeableness									1	0.403***
10. Conscientiousness										1

^*^ *p* < 0.05; ** *p* < 0.01; *** *p* < 0.001

#### Associations of perceived stress, social support, and the Big Five personality traits with emotional exhaustion

Table 3 shows the hierarchical regression analysis of emotional exhaustion after adjusting for covariates. Perceived stress, social support, and the Big Five personality traits together accounted for a large variance in emotional exhaustion (39.0%). Perceived stress (*ß* = 0.577, *p* < 0.001) was statistically significantly associated with emotional exhaustion, accounting for an additional 33.2% of the variance in Step 2. Among social support and the Big Five personality traits in Step 3 and 4, social support (*ß* = 0.117, *p* = 0.002), neuroticism (*ß* = 0.152, *p* = 0.009), and agreeableness (*ß* = -0.181, *p* < 0.001) were individually associated with emotional exhaustion, accounting for an additional 5.8% of the variance in addition to perceived stress. Furthermore, tolerance (range 0.313–0.647) and variance inflation (range 1.546–3.198) did not indicate statistically significant multicollinearity issues.

**Table 3:** Hierarchical regression analyses for perceived stress, social support and Big Five personality traits influencing learning burnout (Emotional exhaustion) in medical students

Variables	Step 1		Step 2		Step 3		Step 4	
	*ß*	*p*	*ß*	*p*	*ß*	*p*	*ß*	*p*
**Covariates**								
Gender	-0.029	0.482	-0.028	0.417	-0.034	0.309	-0.004	0.893
**Perceived stress**			0.577	< 0.001	0.624	< 0.001	0.414	< 0.001
**Social support**					0.117	0.002	0.142	0.001
**The Big Five personality traits**								
Neuroticism							0.152	0.009
Extroversion							-0.015	0.743
Openness							-0.047	0.300
Agreeableness							-0.181	< 0.001
Conscientiousness							0.024	0.669
**F**	0.494	0.482	144.995	< 0.001	101.498	< 0.001	46.027	< 0.001
**R**^**2**^	0.001		0.333		0.345		0.391	
**Adj.R**^**2**^	-0.001		0.331		0.341		0.382	
**R**^**2**^**-changes**	0.001		0.332		0.011		0.046	

*Abbreviations*: *Adj.R*^*2*^ Adjusted R^2^, *ß* Standardized regression coefficient

#### Associations of perceived stress, social support and the Big Five personality traits with cynicism

Table 4 shows the hierarchical regression analysis of cynicism after adjusting for covariates. Perceived stress, social support, and the Big Five personality traits together accounted for a large variance in cynicism (36.8%). Perceived stress (*ß* = 0.543, *p* < 0.001) was statistically significantly associated with cynicism, which accounted for an additional 29.5% of the variance in Step 2. With regard to the Big Five personality traits in Step 4, agreeableness (*ß* = -0.245, *p* < 0.001) was individually associated with cynicism, accounting for an additional 7.3% of the variance in addition to perceived stress. Furthermore, tolerance (range 0.313–0.647) and variance inflation (range 1.546–3.198) did not indicate statistically significant multicollinearity issues.

**Table 4:** Hierarchical regression analyses for perceived stress, social support and Big Five personality traits influencing learning burnout (Cynicism) in medical students

Variables	Step 1		Step 2		Step 3		Step 4	
	*ß*	*p*	*ß*	*p*	*ß*	*p*	*ß*	*p*
**Covariates**								
Gender	-0.122	0.003	-0.120	0.001	-0.118	0.001	-0.079	0.018
**Perceived stress**			0.543	< 0.001	0.531	< 0.001	0.296	< 0.001
**Social support**					-0.030	0.427	0.027	0.523
**The Big Five personality traits**								
Neuroticism							0.100	0.088
Extroversion							-0.012	0.804
Openness							-0.065	0.152
Agreeableness							-0.245	< 0.001
Conscientiousness							-0.045	0.419
**F**	8.749	0.003	129.998	< 0.001	86.820	< 0.001	44.515	< 0.001
**R**^**2**^	0.015		0.310		0.310		0.383	
**Adj.R**^**2**^	0.013		0.307		0.307		0.374	
**R**^**2**^**-changes**	0.015		0.295		0.001		0.073	

*Abbreviations*: *Adj.R*^*2*^ Adjusted R^2^, *ß* Standardized regression coefficient

#### Associations of perceived stress, social support, and the Big Five personality traits with low professional efficacy

Table 5 shows the hierarchical regression analysis of low professional efficacy after adjusting for covariates. Perceived stress, social support, and the Big Five personality traits together accounted for the large variance in low professional efficacy (48.7%). Perceived stress (*ß* = 0.455, *p* < 0.001) was statistically significantly related to low professional efficacy, which accounted for an additional 20.5% of the variance in Step 2. With regard to social support and the Big Five personality traits in Step 3 and 4, social support (*ß* = -0.494, *p* < 0.001), extroversion (*ß* = -0.116, *p* = 0.006), agreeableness (*ß* = 0.098, *p* = 0.008), and conscientiousness (*ß* = -0.363, *p* < 0.001) were individually associated with low professional efficacy, accounting for an additional 28.2% of the variance in addition to perceived stress. Furthermore, tolerance (range 0.313–0.647) and variance inflation (range 1.546–3.198) did not indicate statistically significant multicollinearity issues.

**Table 5:** Hierarchical regression analyses for perceived stress, social support and Big Five personality traits influencing learning burnout (Low professional efficacy) in medical students

Variables	Step 1		Step 2		Step 3		Step 4	
	*ß*	*p*	*ß*	*p*	*ß*	*p*	*ß*	*p*
**Covariates**								
Gender	0.058	0.163	0.060	0.103	0.090	0.005	0.084	0.005
Place of residence	0.025	0.163	0.003	0.947	-0.004	0.917	-0.007	0.862
F Education 1	0.085	0.193	0.067	0.254	0.026	0.605	0.025	0.593
F Education 2	-0.013	0.802	-0.035	0.443	-0.038	0.337	-0.039	0.292
M Education 1	0.056	0.429	0.045	0.474	0.024	0.654	0.007	0.887
M Education 2	0.058	0.256	0.063	0.164	0.031	0.428	0.012	0.735
Exercise	-0.064	0.119	-0.054	0.146	-0.012	0.715	0.009	0.762
**Perceived stress**			0.455	< 0.001	0.259	< 0.001	0.113	0.028
**Social support**					-0.494	< 0.001	-0.319	< 0.001
**The Big Five personality traits**								
Neuroticism							-0.019	0.719
Extroversion							-0.116	0.006
Openness							0.012	0.779
Agreeableness							0.098	0.008
Conscientiousness							-0.363	< 0.001
**F**	2.311	0.025	21.660	< 0.001	47.914	< 0.001	42.980	< 0.001
**R**^**2**^	0.027		0.232		0.429		0.514	
**Adj.R**^**2**^	0.016		0.221		0.420		0.502	
**R**^**2**^**-changes**	0.027		0.205		0.198		0.085	

*Abbreviations*: *F* Father, *M* Mother, *Education 1* Junior high or lower vs. Junior college or higher, *Education 2* Senior high school vs. Junior college or higher, *Adj.R*^*2*^ adjusted R^2^, *ß* Standardized regression coefficient

## Discussion

The main purpose of this study was to discuss the effects of perceived stress, social support, and the Big Five personality traits on learning burnout among Chinese medical students during the COVID-19 pandemic. Some studies have indicated that the phenomenon of learning burnout still exists among medical students. At the cognitive level, medical students often face high levels of academic stress, especially during the pandemic [15]. During the epidemic prevention and control period, medical students were not allowed to participate in clinical probation and delayed their medical practitioner examinations. Meanwhile, medical students faced increasing employment pressures [40]. At the social level, Chinese universities adopted closure and control measures for nearly two years, prompting students to reduce their activities, maintain long-term social distancing, and reduce socialization. At the individual characteristic level, medical students with different personality characteristics had different effects on the level of learning burnout during the COVID-19 pandemic. All these factors are helpful in improving the learning burnout levels of medical students. This study focused on studying the learning burnout of medical students in their third year of university because the excessive load of clinical subjects brings different pressures and lead to learning burnout.

The demographic factors of learning burnout were analyzed in three medical colleges, and it was found that gender, place of residence, and parental educational status had an impact on learning burnout. The results indicate that male medical students were more susceptible to cynicism than female medical students. However, one study showed that female students were more likely to experience burnout than male students [41]. The results of this study are consistent with those of Lin et al. [42]. A possible reason for this is that male students have weak self-control and are easily disturbed by external temptations. They desire success, doubt the meaning and usefulness of learning, become disinterested, and shift their interest in learning. Furthermore, they were addicted to online games to get a sense of achievement, were late for class and left early, skipped class, used mobile phones in class, slept, played games, and misbehaved.

The COVID-19 pandemic, in its multiple impacts, has exacerbated pre-existing inequalities in several domains including income and employment, education, and family, and health. Inequalities (such as economic, social, educational, age, gender, occupational, and geographic) have worsened in the short term and are likely to persist in the future [43, 44]. The quarantine measures have caused financial uncertainties, and brought “social and economic life to a near stop”, particularly for those living in developing countries [45, 46]. In addition, the income gap between urban and rural residents in China has widened significantly. The results showed that students who lived in villages were more likely to have low professional efficacy than those who lived in cities. This result is consistent with previous research [47, 48], which might be because students in villages need to learn online in the context of the epidemic. Furthermore, they faced additional financial pressure from their families, which made successfully completing online learning difficult for some students. This leads to lower professional efficiency, which might lead to more learning burnout.

Educational attainment was used as an indicator of socioeconomic status [49]. A 30% difference in educational attainment based on socioeconomic status lays bare the heavier burden of low-income groups [50]. Studies conducted abroad have shown a close relationship between family socioeconomic status and learning burnout [51]. The main targets of family socioeconomic status are the family’s economic income and parents’ educational level and occupation [51]. The results suggest that students whose parents had a junior high education or lower were more likely to have low professional efficacy than those whose parents had a junior college education or higher. The possible reason is that parents with low education levels could hardly be role models for students, and it was difficult for them to give certain encouragement and support to their children’s learning. Additionally, students have unequal access to equipment and other resources. Students from families with a lower socioeconomic status have fewer resources [51]. These inequalities are in line with the findings of the Portuguese National Board of Education report, where 21% of the teachers answered that more than 30% of their students were affected by the absence of digital devices at home [52]. Another study in the US shows that lower income and ethnic minority families experienced greater stress related to income loss and financial costs from April to June 2020, while higher income and White families were more stressed about distance learning [53]. Generally, students from these families want to finish their studies as early as possible and gain less sense of achievement from their studies. Families with high socioeconomic status schedule more cultural and social amusement for their children, which in turn leads to a reduction in learning burnout. Therefore, family influence on students may lead to lower professional efficacy.

The effects of perceived stress, social support, and Big Five personality traits on medical students’ learning burnout was analyzed. The results of hierarchical linear regression analysis showed that perceived stress and social support had a significant effect on learning burnout. The results also showed that perceived stress was the strongest predictor of emotional exhaustion and cynicism among all students and had a positive effect on emotional exhaustion, cynical attention and low professional efficacy. This result was consistent with that of previous studies [7, 12, 54]. Hendrix et al. [55] also noted that higher perceived stress was associated with higher emotional exhaustion, personality dissociation, and lower levels of personal accomplishment. The reason may be that some factors of stress can cause students to feel fatigued, have a cynical and detached attitude towards their studies, and feel incompetent. This study focused on third-year undergraduate students, who may be facing increasing pressure from professional clinical courses, especially in the context of epidemics, leading to increased burnout.

Additionally, the findings showed that social support had a negative impact on low professional efficacy. This result is consistent with that of most studies [2, 56, 57]. Studies have pointed to life satisfaction as a key factor underlying psychological assessment or mood, which helps explain why increased social support is related to lower learning burnout. Students who receive more support are more easily satisfied with their lives, which in turn may reduce learning burnout [2]. Although life satisfaction was not measured in this study, this may explain why social support has a negative effect on learning burnout. Other studies have shown that social support has a protective effect against burnout symptoms among medical students [57, 58]. Adequate social support is likely to produce a positive outlook on life, regardless of realistic material resources, which will help them overcome low professional efficacy. Social support can not only transmit its effects indirectly through other factors related to learning burnout, but also directly affect learning burnout [59, 60]. This substantiates the theoretical framework that social support influences both internal and external aspects of learning burnout. However, contrary to expectations, social support has a positive effect on emotional exhaustion. According to a previous study, social support is negatively correlated with emotional exhaustion. When individuals think, they engage in supportive transactions. However, the element of shared reflection inhibits the positive effects of social support on outcomes related to emotional exhaustion [61]. The blunting effect of positive social support makes sense from a strictly psychological perspective, a finding that may be replicated in physiological outcomes. The fact that the person participated in communal rumination, regardless of their specific role, was sufficient to reduce the positive effect of social support on emotional exhaustion in this sample.

Furthermore, in each dimension of the Big Five personality traits, agreeableness was most closely related to emotional exhaustion and negatively affected emotional exhaustion and cynicism. Such people are more likely to receive social support and have their problems solved [62]. However, in this study, students who scored high on agreeableness were prone to low professional efficacy. Students with high agreeableness scores were more concerned, considered problems from the perspective of others, were easily influenced by others, and had a lower sense of achievement in learning. To the best of our knowledge, this is the first study to test the relationship between agreeableness and low professional efficacy among clinical medical students during the pandemic. The sample size was then increased to further verify the results. The Cronbach’s alpha of agreeableness was 0.582 in this study, which was in line with an empirical study of Philippine college students (Cronbach’s a = 0.6) [17]. Therefore, related inferences should be drawn prudently.

The findings suggest that extraversion has a negative impact on low professional efficacy. Extraversion refers to social skills and agency and the tendency to experience positive emotions. Extraverted college students may be more willing to ask others for help when they encounter learning disabilities, thereby reducing the occurrence of low professional efficacy. Furthermore, the results showed that neuroticism, a personality trait that is interconnected with a tendency to experience negative emotions and anticipate the worst situation, positively affected emotional exhaustion. Such people also tend to underestimate their self-expression. Students with high neuroticism appear to be more likely to experience emotional exhaustion because of their susceptibility to stress and difficulties in dealing with it. Additionally, students who do not fit in may experience more burnout because they are picky and determined [63]. These attributes may hinder them from building supportive relationships with others, thus making them more prone to emotional exhaustion. These results are partly supported by the finding that neuroticism is one of the causes of emotional exhaustion [64]. Hochwälder et al. [65] found that personality traits explained 7% of the variance in emotional exhaustion, whereas Bakker et al. [18] found that neuroticism was the only predictor of emotional exhaustion, accounting for 13% of the variance. Students with neurotic personalities had an experiential preference for negative things. Once failure is encountered, it is easy to develop learned helplessness, which leads directly to emotional exhaustion.

Among all the variables, conscientiousness had the strongest correlation with low professional efficacy and a negative impact on low professional efficacy. This result is similar to that of a previous study [64]. Conscientiousness appears to be a key personality trait associated with individual achievement [64]. Consistent with the present study’s findings, individuals with lower levels of conscientiousness are at risk of increased personal effectiveness in situations of role conflict and higher workloads [66]. People who score high on conscientiousness are more organized, plan ahead, and think more carefully. This type of person may not be overworked and tends to adhere to learning tasks [67].

As mentioned above, perceived stress, social support, and the Big Five personality traits together accounted for large variances in emotional exhaustion (39.0%), cynicism (36.8%), and low professional efficacy (48.7%). This provides further evidence of the influence of perceived stress, social support, and the Big Five personality traits on learning burnout. Furthermore, in addition to these types of factors, there may be other factors that influence learning burnout. Further research is required to clarify this aspect.

### Limitations

This study had several limitations. First, the cross-sectional results cannot infer causality or the underlying mechanisms between learning burnout and related factors. Due to the lack of a longitudinal design, this study was unable to track the variables of students and their medical education processes. Future research projects should use longitudinal designs to establish causal relationships between variables. Second, the participants in this study were all from three medical schools in Liaoning Province, China, which may limit the generalizability of the findings. Further studies are needed to examine whether the results of the present study are suitable in different cultural contexts and for other samples. Third, all data were self-reported, which may have led to a bias. Participants may have underestimated or overestimated the learning burnout of Chinese medical students and their relationship with social support, perceived stress, and the Big Five personality traits. An in-depth evaluation should be conducted to identify learning burnout, which could provide a more specific understanding of learning burnout among medical students. Fourth, the participants’ opinions or feelings can only represent a point in time. Therefore, long-term follow-up studies should be conducted during the COVID-19 pandemic. Fifth, the NEO-FFI agreeableness Cronbach’s alpha value was quite low (0.582), and thus, the current findings should be interpreted more carefully. Finally, future studies should also assess other factors that may influence burnout episodes (such as consistency and optimism) or the inverse relationships between study variables.

## Conclusions

This study found that perceived stress had a positive impact on burnout and was the strongest indicator of learning burnout. Social support had a positive effect on emotional burnout and negative effect on low professional efficacy. Neuroticism had a positive effect on emotional burnout; extraversion and conscientiousness had a negative effect on low professional efficacy; and agreeableness had a negative effect on emotional burnout, cynicism, and low professional efficacy. Different interventions should be carried out according to students’ perceived pressure, social support, and personality characteristics to solve the problem of learning burnout. The results of this study can be used to help educators, administrators, psychologists, and other health professionals develop necessary interventions for students suffering from learning burnout.

## Data Availability

The datasets used and/or analysed during the current study available from the corresponding author on reasonable request.

## Abbreviations

ANOVA: Analysis of Variance
COVID-19: Coronavirus Disease 2019
MBI: Maslach Burnout Inventory
MBI-SS: Maslach Burnout Inventory-Student Survey
NEO-FFI: NEO Five-Factor inventory
PSS-10: Perceived Stress Scale
PSSS: Perceived Social Support Scale
